# The experiential basis of compatibility effects in reading-by-rotating paradigms

**DOI:** 10.1007/s00426-022-01663-1

**Published:** 2022-02-27

**Authors:** Francesca Capuano, Berry Claus, Barbara Kaup

**Affiliations:** 1 Department of Psychology, University of Tübingen, Schleichstr. 4, 72076 Tübingen, Germany; 2 Department of German Studies and Linguistics, Humboldt-Universität zu, Berlin, Germany

**Keywords:** Action-sentence compatibility, Embodiment, Individual differences, Language, Simulation view

## Abstract

The current study originates from inconsistent findings within the framework of embodied language processing, specifically in the reading-by-rotating literature: whereas some studies report a match advantage (e.g., Zwaan and Taylor (J Exp Psychol 135:1, 2006)), i.e., shorter reading times when the direction of a linguistically conveyed manual rotation matched rather than mismatched the direction of an actually to be performed manual rotation Claus (Acta Psychol 156:104–113, 2015) found a mismatch advantage. The current study addresses two explanations that were previously discussed as potentially responsible for this inconsistency: on the one hand, differences in the knob devices employed; on the other hand, differences in the perspectives adopted by the readers depending on the number of characters involved in the narratives. Concurrently, the study exploits individual differences in motoric experience to explore the experiential basis of action-sentence compatibility effects. The results are inconclusive with respect to the two explanations. However, in their overall picture, they contribute suggestive considerations for the ongoing debate on action-simulation effects by pointing to the potential role of interindividual variation in motoric experience.

## Introduction

Traditional approaches to cognition see language as a mental system manipulating abstract, amodal and arbitrary symbols by means of grammar rules (Fodor, 1975; Kintsch, 1988; Pylyshyn, 1984). In this perspective, no relevance is given to connections between mental representations of linguistic meaning and representations of our bodily experiences.

Theories of embodiment on the other hand postulate a representational overlap between perception, action and cognition: sensorimotor traces formed during our physical interaction with the environment are reactivated and newly combined to yield mental simulations that make up cognition. These traces are experiential, multimodal and interconnected via co-occurrence learning mechanisms. As part of cognition, language processing itself might be grounded in perception and action, and exploit the same kind of representations as those involved in perception/action (Barsalou, 1999; Glenberg, 1997; Zwaan, 2004). Specifically, embodied-simulation views of language comprehension suggest that understanding words and sentences involves mentally simulating the actions, events and referents being described through the reactivation of experiential traces (Zwaan & Madden, 2005). For example, understanding a sentence like *John pets the dog* would reactivate perceptual traces of direct experience with dogs, as well as motor traces of petting.

Whereas the limitations of the functional role of such simulations are still under discussion (Fischer & Zwaan, 2008; Kaup et al., 2015), the literature includes evidence claiming support for embodied-simulation views. fMRI studies have highlighted physical overlap of brain patterns active during language comprehension with the ones active during direct experience of the linguistic content. For example, González et al. (2006) showed increased brain activity in the olfactory region when participants read words with referents associated with a strong olfactory experience, such as *cinnamon*. Additional support is presumably provided by behavioural studies dealing with so-called *action-sentence compatibility effects* (Glenberg & Kaschak, 2002), although there is some debate about the extent to which the original effect can be replicated (see, e.g., Papesh, 2015). These studies show that the comprehension of linguistic material describing actions affects actual physical movements of the reader that are relatable to the direct performance of these action, in a way that hints at the reactivation of the corresponding motor traces. One variant of this paradigm is the reading-by-rotating paradigm introduced by Zwaan and Taylor (2006), who uncovered a compatibility effect between the content of a sentence and the actual movement participants performed during reading. In Zwaan and Taylor (2006) participants read sentences such as (1) frame by frame, proceeding from one frame to the next by rotating a knob in either a clockwise or counterclockwise direction. Each sentence contained a critical frame implying a hand rotation movement (e.g., *turned down* in (1)) that is usually performed in either a clockwise or counterclockwise direction (e.g., counterclockwise for *turned down*).

1. He / realized / that the / music / was / too loud / so he / turned down / the / volume.
                                      [The slashes indicate the frame boundaries.]

Reading times for the critical frames were shorter when the described actions involved a direction of rotation matching the required direction of rotation of the knob, compared to when the two directions were opposite. The finding of a match advantage was reproduced in further studies employing the reading-by-rotating paradigm (Taylor & Zwaan, 2008; Taylor et al., 2008; Zwaan et al., 2010).

However, a study applying the reading-by-rotating paradigm on verb gapping with sentences such as (2) produced the opposite pattern of results, with a reading time advantage for mismatching rotation directions in the frame with the overt verb (e.g., *opens a lemonade bottle*) as well as in the frame with the gapped verb (e.g., *a juice bottle*) (Claus 2015).

(2) Tina / opens a Fanta bottle / on the balcony / and Adrian / a juice bottle / in the children’s room.
                          [Translated from German.]

This inconsistency seems to count against an embodied simulation view of language comprehension, as it might lead to the suspicion that compatibility effects arise arbitrarily and inconsistently across studies.

Claus (2015) discussed how the opposite findings can be reconciled within the embodiment framework by considering differences in the experimental task demands. One account pertains to differences in the specific knob turning procedures. Specifically, in Zwaan and Taylor (2006) participants were instructed to rotate the knob to trigger each frame change and to keep holding it in the current position before proceeding to the next frame. Only at the end of each item could they release the knob, which due to the presence of springs would come back to the original central position. In Claus (2015) the knob (also containing springs) was to be released back to the central position after each frame turn. The two different procedures could potentially explain the mismatching results, as they might have led to differences in the relative temporal dynamics of mental simulation and motor planning. Possibly, the necessity to keep holding the knob in Zwaan and Taylor (2006) resulted in temporal overlap of the two processes, whereas in Claus (2015) motor planning might have succeeded mental simulation. This could be decisive if temporal succession of mental simulation and motor planning, as opposed to overlap, inhibits rather than facilitates a matching action. The idea is supported by studies pointing to the lack of a compatibility effect when motor instructions are given after sentence presentation (e.g., Borreggine & Kaschak, 2006; Diefenbach et al., 2013; Kaschak & Borreggine, 2008; but see de la Vega et al., 2013 for different findings). An alternative account of the opposite patterns of results relates to a difference in the materials. Each experimental item of Zwaan and Taylor (2006) involved only one main character, whereas there were two main characters in each experimental item of Claus (2015). This difference in number of characters might have led to a difference in perspective adoption (Franklin et al., 1992). With one character, the default might be an internal, character-centered perspective, yielding a simulation of performing the described actions. However, with two characters, comprehenders might adopt an external, “en face” perspective (cf. Sato et al., 2012) and simulate the observation, rather than the execution, of described actions.

The current study aims at exploring the validity of the two accounts of the conflicting findings. We used the exact same device as in Claus (2015), but our materials always described only one character. Thus, if the device is the critical factor, a mismatch advantage should be observed as in Claus (2015). If on the other hand the number of characters is crucial, a match advantage should be observed as in Zwaan and Taylor (2006).

**Fig. 1:**
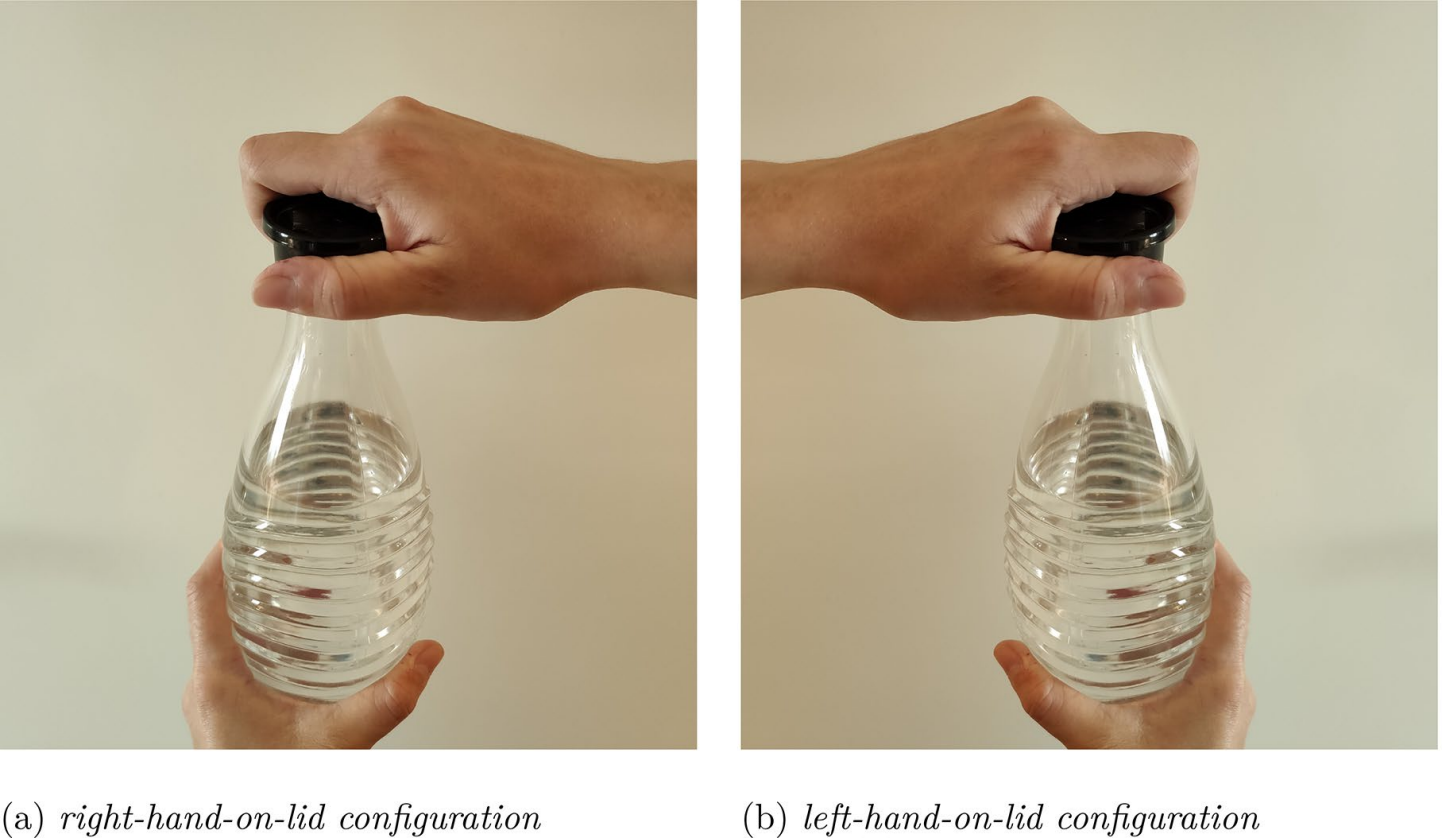
Hands configurations in the actions of opening/closing bottles

However, there is another major difference in the materials between the studies by Zwaan and Taylor (2006) and Claus (2015). Whereas all the target actions in Claus (2015) were opening/closing actions of objects with a lid (e.g., bottles, jars, tubes), the sentences in Zwaan and Taylor (2006) were more mixed (e.g., sharpening a pencil, screwing in a light, opening a jar, dimming the lights; with only three out of 16 items involving objects with a lid). This difference in the materials may have been crucial when considering the possibility of individual differences in actually performing the described actions. Most of the target actions in the material of Zwaan and Taylor can be assumed to be uniformly performed in one direction with one hand (or with both hands, e.g., turning left while driving). This is different for the actions in the materials of Claus: opening/closing objects with a lid often involves the simultaneous rotation of both hands in opposite directions. For instance, if the right hand is on the lid of an object and the left hand is on the base of the object, then the right hand turns clockwise for closing and counterclockwise for opening, whereas the left hand—positioned on the base of the container—turns counterclockwise for closing and clockwise for opening; and vice versa if the left hand is on the lid (see Fig. 1). In this matter, it seems conceivable that individual preferences for the use of a hand over the other in rotating lids (independently of the participant’s handedness) might be reflected on a match or a mismatch advantage.

In the study by Claus, all participants were right-handed and assumed to use the right hand on the lid when opening/closing objects with lids: the levels match and mismatch were assigned to the experimental conditions (described action: open/close x knob turning direction: counterclockwise/clockwise) according to the manual rotation of the hand on the lid (match: open + counterclockwise and close + clockwise; mismatch: open + clockwise and close + counterclockwise) and the participants were explicitly instructed to rotate the knob only with the right hand. However, the above considerations suggest that this assignment of the match and mismatch levels might not be generally appropriate. Only for participants who preferably have their right hand on the lid does the right-hand knob turning in the match/mismatch conditions of the experiment indeed match/mismatch their right hand rotations when actually performing the described opening or closing actions. However, for participants who preferably have their right hand on the base, the mapping of match and mismatch is actually reversed, because the direction of the rotation of the hand on the base is opposite to that of the hand on the lid (match: open + clockwise and close + counterclockwise; mismatch: open + counterclockwise and close + clockwise). Thus, for these participants, the right-hand knob turning in the match conditions of the experiment mismatches rather than matches their right hand rotations when actually performing the described actions, and vice versa for the mismatch conditions: for example, when reading about an opening action, their simulation might involve a counterclockwise turning of the left hand but a clockwise turning of right hand, such that the mismatch condition, requiring a clockwise turning of the knob, mismatches the simulation for the left hand but actually matches the simulation for the right hand (the hand that performs the actual knob turning) resulting in a mismatch advantage. Hence, for the former group of participants (right hand on lid), a match advantage is expected, reflecting the pattern of movements of the right hand on the lid, whereas for the latter group of participants (right hand on base), a “mismatch” advantage is expected, reflecting the pattern of movements of the right hand on the base of the containers.

According to this line of reasoning, the mismatch advantage observed by Claus (2015) might be due to the possibility that the majority of participants in her study preferably use their right hand on the base when opening/closing containers with a lid. To pursue some insight with regard to this explanation, we included an assessment of participants’ hand preferences in the present experiments.

## Experiment 1

### Method

#### Participants

Eighty right-handed native German students from the University of Tübingen took part in the experiment (15 male and 65 female, age ${\text {mean}}=23.29$ and ${\text {sd}}=5.75$). They gave informed consent in written form and received either partial course credit or a monetary reimbursement (8 euros/h) for their participation.

#### Materials

The materials consisted of short narrative texts in German, very similar to the ones from Claus (2015). Each text was composed of three sentences. The first sentence served as scene setting and introduced the single main character of the text. Differently from Claus (2015), the second sentence always described only one action or event. Finally, the third sentence concluded the narrative.

All the sentences were segmented by intuitively natural boundaries for the purpose of frame-by-frame presentation. In the case of the experimental items, the second, critical sentence always had the same structure, and was divided into three frames: the first frame included the pronoun *he* or *she*, the second frame (the critical frame) included the verb *öffnet* (opens) or *schließt* (closes) followed by a noun phrase describing an object with a lid that can be opened by manual rotation, the third frame was always a prepositional phrase (potentially the spillover effects region), as in (3):

(3) *Er/ öffnet ein-e Fanta -flasche/ auf d-em Tisch. *
  He/ opens a-acc Fanta bottle/ on the-dat table.
  ‘He opens a Fanta bottle on the table.’

The 24 different noun phrases denoting objects with a lid (10 kinds of bottles, five kinds of jars, five kinds of tubes, four others such as *petrol can*) employed in Claus (2015) were selected as experimental items. Each item was presented once in a version with *opens* and once in a version with *closes*. For each experimental item, the two versions constitute two different narrative texts with different main characters and different scene settings and conclusions congruent with the opening action or the closing action described in the respective version of the second sentence. The critical frame of the second sentence was identical in the two versions, except for the verb. The gender of the narrative main character was counterbalanced across versions and objects. An example of a complete narrative text of one experimental item can be found in (4) (translated into English, with the original German text in parentheses). See the Appendix A for the complete list of critical sentences.

(4) **Clockwise:** It’s about three in the morning and bartender Jessica finishes her shift in the bar. She closes an amaretto bottle behind the counter. Then she rinses the last glasses. (Es ist gegen drei Uhr früh, und die Barkeeperin Jessica beendet ihre Schicht in der Bar. Sie schließt eine Amarettoflasche hinter dem Tresen. Dann spült sie noch die letzten Gläser.)
  **Counterlockwise:** The bar owner Ben stands behind his bar and welcomes one of his regular customers who has just arrived. He opens an amaretto bottle on the counter. Then he mixes an amaretto cocktail for the regular customer. (Der Barbesitzer Ben steht hinter seiner Bar und begrüßt eine seiner Stammkundinnen, die gerade gekommen ist. Er öffnet eine Amarettoflasche an der Theke. Dann mixt er einen Amaretto-Cocktail für die Stammkundin.)

Fifty-two fillers and four practice texts were constructed. The filler and practice texts were structurally similar to the experimental texts, but the second sentence described actions other than the opening/closing of objects with a lid. Twenty-six of the fillers and two of the practice trials were accompanied by a yes/no comprehension question to control for participants’ attentive accomplishment of the reading task.

#### Apparatus

The exact same knob device from Claus (2015) was employed as input device for the self-paced reading task (reading-by-rotating paradigm). The knob has a diameter of 4.5 cm and is mounted on a box that allows it for rotation on the horizontal plane. A rotation of approximately 60 degrees logs the reaction time and prompts the successive frame. The knob contains springs that bring it back to the central position once it is released.

#### Design and procedure

The experiment was divided in two blocks. Direction of knob turning was counterbalanced and manipulated within subjects. Each participant was assigned a direction of knob rotation for the first block (clockwise vs. counterclockwise), that was switched in the second block. Each block started with two practice trials (one with and one without a comprehension question at the end), in order for the participants to get accustomed both to the direction of rotation and to the task.

Every participant saw all 24 experimental items both in the version with *opens* and in the version with *closes* (24 in each block, corresponding to the 24 different objects with a lid). The same amount of items in the *closes* and in the *opens* versions was presented in each block. Approximately the same number of objects of the same kind was presented in each version in each block (i.e., five bottles in each version, two jars in one version and three jars in the other version, three tubes in one version and two tubes in the other version, two of the other objects in each version). Each participant saw all fillers. Each block contained the same amount of fillers without a question, fillers with a question plus a *yes* answer, and fillers with a question plus a *no* answer. The presentation of the experimental texts and filler texts was pseudo-randomised, such that no more than two experimental texts would appear consecutively, and no more than two experimental texts in the same version would follow each other (either directly follow each other or being interrupted by ore or more fillers). Out of every 10 texts presented, at least two were fillers accompanied by a question.

The experiment was run individually in the laboratory. Only right-handed participants were recruited, and their handedness was self-assessed once again before the start of the experiment. Participants sat in front of a pc screen, on which the texts were presented in 9 pt Courier New. They were explicitly instructed to use their right hand to rotate the knob. Questions at the end of the fillers were announced by three question marks and could be answered with a yes or no key on the keyboard with the left hand. Each frame’s reading time was defined and logged as the interval between presentation of frame and successive knob turning. Participants were instructed to read the texts carefully at their natural pace.

At the end of the reading task, participants’ preference for the use of one hand over the other in rotating lids was self-assessed via answering the question “Which hand do you usually use on the lid to open/close bottles?”.

### Results and discussion

Participants who answered to the comprehension questions at no more than chance level were excluded from the analysis ($n=1$). The data were analysed with linear mixed-effects models. For the sake of replicability though, we additionally conducted an analysis with ANOVAs and t-tests akin to Claus (2015): this constitutes a more conservative strategy, because the data processing and assessment procedures were established a priori. The results of this analysis are very similar to the results of the main analysis described below. For the results of the more conservative analysis, see Appendix B.

The data cleaning for the analysis employing linear mixed-effects models was the following: RTs smaller than 100 ms were excluded, after which RTs deviating more than 2.5 standard deviations from the mean of the corresponding condition (Match x Frame x Subject) were rejected. In total, 4% of the data was excluded from the analysis.

The original analysis from Claus (2015) involved a test of the Match by Frame interaction. Accordingly, the analysis in the appendix includes the interaction. However, a power analysis reveals that a sample size of 967 participants is required to uncover a small effect (Cohen’s $f = 0.1$) with 80% power (Faul et al., 2007). Therefore, we here report a simplified analysis without the factor Frame.

**Fig. 2:**
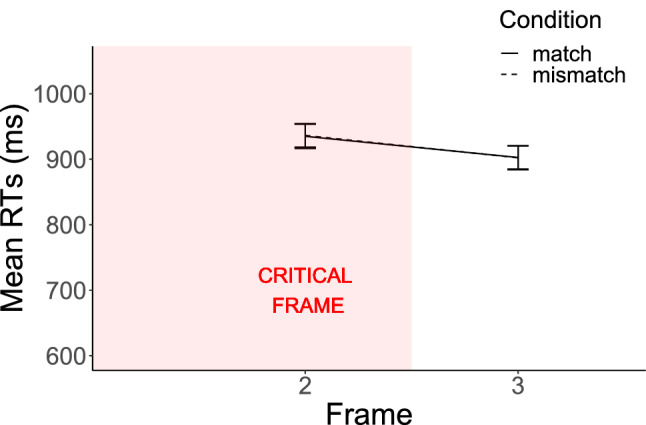
Experiment 1: overall results. Mean RTs in the match and mismatch conditions on the three frames of the critical sentence. Error bars represent 95% confidence intervals of the means

Because we expected a compatibility effect, if any, to appear from Frame 2 (the critical frame) onwards, we still tested for a main effect of Match on Frames 2 and 3. By comparing Model 1

1 $$\begin{aligned} {\text {RT}} \sim {\text {Match}} + (1 |{\text {Item}}) + (1 |{\text {Subject}}) \end{aligned}$$

with Model 2

2 $$\begin{aligned} {\text {RT }}\sim 1 + (1 |{\text {Item}}) + (1 |{\text {Subject}}) \end{aligned}$$

on the relevant data, no effect of Match was found on Frame 2 (${\chi }^2(1)=.01$, $p=0.90$), nor on Frame 3 (${\chi }^2(1)<0.01$, $p=0.95$). These results neither replicate the results of Zwaan and Taylor (2006) nor those of Claus (2015) (see Fig. 2).

In a next step, we tested our hypothesis that participants may exhibit differential reading time patterns on the critical frame and possibly on the subsequent frame, depending on which hand they usually use on the lid. We analyzed the data by including Hand-on-Lid as an additional factor (56 participants stated a preference for the right hand on the lid, 23 for the left hand).

The Match x Hand-on-Lid interaction (Model 3 vs. Model 4) was significant on both Frame 2 (${\chi }^2(1)=8.20$, $p<0.01$) and Frame 3 (${\chi }^2(1)=4.37$, $p<0.05$):

3 $$\begin{aligned} {\text {RT}}\sim & {} \mathrm{{Match}} \times {\text {Hand.on.Lid }}+ (1 |{\text {Item}}) + (1 |{\text {Subject}}) \end{aligned}$$

4 $$\begin{aligned} {\text {RT}}\sim & {} \mathrm{{Match}} + {\text {Hand.on.Lid}} + (1 |{\text {Item}}) + (1 |{\text {Subject}}) \end{aligned}$$

A power analysis of this interaction reveals that a sample size of 80 participants is sufficient to reach 82% power. Although this power analysis was conducted post-hoc, it can serve to corroborate the sample size employed in Experiment 2 ($n=80$). Vice versa, the same power analysis on Experiment 2 reaches 81% power and a posteriori corroborates the sample size employed in Experiment 1.

Next, we analysed more closely the Match x Hand-on-lid interactions observed for Frames 2 and 3. Here we tested the specific hypothesis that a match advantage should occur for the right-hand-on-lid group and a mismatch advantage for the left-hand-on-lid group. We, therefore, conducted separate analyses for the two sets of participants.

**Fig. 3:**
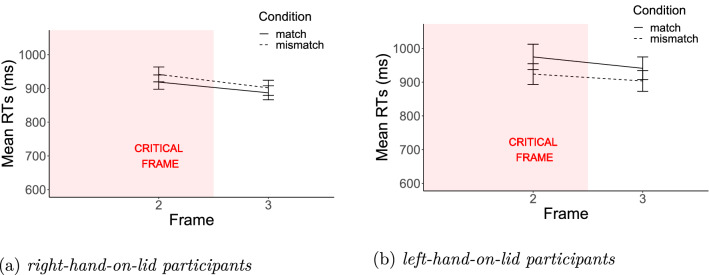
Experiment 1: results by motoric experience. Mean RTs for participants accustomed to rotate lids with the left hand (left panel) and for participants accustomed to rotate lids with the right hand (right panel). Error bars represent 95% confidence intervals of the means

On Frame 2, the group of participants who use the right hand on the lid (9 male and 47 female, age $\mathrm{{mean}}=23.52$ and $\mathrm{{sd}}=5.55$) displayed a significant match advantage (Model 1 vs. Model 2) when evaluated directionally with one-tailed t tests (${\chi }^2(1)=2.80$, $p=0.09$), whereas participants who use the left hand on the lid (6 male and 17 female, age $\mathrm{{mean}}=22.48$ and $\mathrm{{sd}}=6.26$) displayed a significant mismatch advantage (${\chi }^2(1)=5.38$, $p<0.05$). On Frame 3, the effect of Match was only significant for the left-hand-on-lid group (right: ${\chi }^2(1)=1.14$, $p=0.28$; left: ${\chi }^2(1)=3.24$, $p=0.07$). See Fig. 3 for the results of Experiment 1 by motoric experience.

These results thus align with the idea that a compatibility effect holds between the content of a sentence describing an action and the way participants effectively perform the action. We conducted an additional experiment using a slightly different paradigm to investigate whether similar results would be obtained again and at the same time find out whether the results would generalize to a slightly different setting.

## Experiment 2

In Experiment 1, to advance from frame to frame, participants rotated the knob in the same direction throughout each of the two experimental blocks. This might have led to a rather automated performance of the movement. In Experiment 2 the direction of knob turning changed on a frame-by-frame basis, as participants had to choose between the two directions of turning depending on the font color in which the frame was presented.

### Method

#### Participants

A new sample of 80 right-handed native German students from the University of Tübingen who had not participated in Experiment 1 took part in the experiment (20 male and 60 female, age $\mathrm{{mean}}=22.76$ and $\mathrm{{sd}}=4.16$). They gave informed consent in written form and received either partial course credit or a monetary reimbursement (8 euros/h) for their participation.

#### Materials

The same materials were employed as in Experiment 1.

#### Apparatus

The same knob device was employed as in Experiment 1.

#### Design and procedure

The procedure was similar to the one for Experiment 1. However, this time each frame was displayed in either one of two colours (blue or orange). The colour indicated which direction of rotation was required to proceed to the next frame. The experiment was run in one block, preceded by four practice trials. The colour-direction association was counterbalanced across participants. The colour of the frame preceding the critical frame was counterbalanced across items. As in Experiment 1, each participant saw all 24 experimental items (once in the version with *opens* and once in the version with *closes*) and all the fillers. Again we tried to control for object type by presenting at best the same number of objects of the same kind in each condition. Items presentation was pseudo-randomised in the same way as in Experiment 1, but with the additional constraint that the clockwise and counterclockwise context versions of the same item were at a distance of at least 10 other experimental texts.

At the end of the experiment, participants were asked to state their preference for the use of the right or the left hand on the lid of bottles, as in Experiment 1. This time they were additionally handed an actual bottle, with the instruction that they could use it to simulate the actions of opening and closing, to better assess their preference before communicating it to the experimenter.

### Results and discussion

**Fig. 4:**
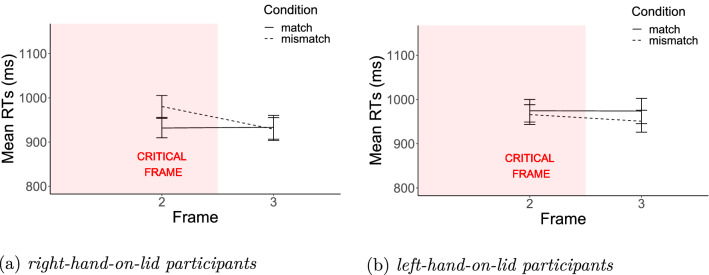
Experiment 2: results by motoric experience. Mean RTs for participants accustomed to rotate lids with the left hand (left panel) and for participants accustomed to rotate lids with the right hand (right panel). Error bars represent 95% confidence intervals of the means

The data cleaning procedure was the same as in Experiment 1. One participant was excluded from the analysis, because the accuracy of their responses to the comprehension questions was at chance level. Altogether, 4% of the data were discarded. Figure 4 shows the mean RTs in the three frames separately for the two groups of participants (36 left-hand-on-lid vs. 43 right-hand-on-lid participants).

As in Experiment 1, we conducted separate analyses for the relevant frames as we expected Match x Hand-on-lid interactions only from Frame 2 onwards. There was a significant interaction on Frame 2 (the critical frame) (${\chi }^2(1)=7.54$, $p<0.01$), but not on Frame 3 (${\chi }^2(1)=.79$, $p=0.37$). We analyzed the observed Match by Hand-on-Lid interaction on Frame 2 more closely by performing separate analyses for the two groups of participants. For the group that prefers using the right hand on the lid (11 male and 32 female, age $\mathrm{{mean}}=23.42$ and $\mathrm{{sd}}=4.94$) a significant match advantage was found on the critical frame (${\chi }^2(1)=11.49$, $p<0.001$). For those who use the left hand on the lid (9 male and 27 female, age $\mathrm{{mean}}=22.03$ and $\mathrm{{sd}}=2.93$) there was a numerical mismatch advantage on the critical frame, but this advantage failed to reach significance (${\chi }^2(1)=.29$, $p=0.59$).

Again, we observed a difference between the two participant groups who differ in their motor experiences when opening/closing bottles. While a significant match advantage was found for those participants who use the right hand on the lid, no significant difference was observed for those participants who use the left hand on the lid.

## General discussion

The initial aim of the current study was to explore the role of different knob turning procedures and different number of characters involved in the narratives in explaining conflicting findings of studies employing the reading-by-rotating paradigm. Specifically, Zwaan and Taylor (2006) found shorter reading times for sentence frames describing hand rotations, when the implied direction of rotation (clockwise or counterclockwise) matched the direction of hand rotation that the participants were instructed to perform immediately afterwards. In contrast, Claus (2015) found a mismatch advantage.

In our study we used the exact same knob device that was used in Claus (2015), and we presented items describing the opening and closing of the same objects as employed in the materials of Claus (2015). We changed the number of characters described in the texts, such that the actions would involve only one character (vs. two characters in, Claus, 2015). If the inconsistency in the two studies were attributable to the difference in number of characters, an overall match advantage was to be expected. If, instead, the difference in the knob turning procedure was crucial, an overall mismatch advantage was to be expected. The overall results show no significant difference for the match vs. mismatch condition on the critical frame.

An additional difference in the materials of the two studies appeared to be potentially relevant: all target actions of Claus (2015) were actions of opening/closing containers with a lid, which often involve the simultaneous rotation of both hands in opposite directions, whereas the target actions of Zwaan and Taylor (2006) were more heterogeneous and mostly implied only one direction of rotation. One of the fundamental assumptions of embodied simulation views of language comprehension is that mental simulations involve the reactivation of individual experiential traces. If the experience of the participants differs in terms of being accustomed to rotate lids with the right vs. left hand, then differential patterns of motor trace reactivation should be expected. By proceeding along this line of reasoning, we explored the experiential basis of action-sentence compatibility effects by inquiring individual motor experiential differences.

Experiment 1 indeed showed a different pattern of results depending on participants’ hand preferences when opening/closing bottles. A match advantage was found for participants who use the right hand on the lid, and a mismatch advantage for those that use the left hand on the lid. This translates in a compatibility effect between the action described in the sentence and the action performed by the participants, as they are accustomed to perform it; when defining match and mismatch according to participants’ self-assessed hand preferences, both groups showed a match advantage. Experiment 2 replicated the finding for the right-hand-on-lid group, but the compatibility effect did not reach significance level for the left-hand-on-lid group. The non-significant compatibility effect for the group that uses the left hand on the lid is maybe attributable to reduced statistical power with respect to this group. However, more in general, the compatibility effect elicited in the two studies might simply be more stable for right-handers who use the right hand on the lid. In fact, right-handers who prefer the left hand on the lid might be less consistent in the use of the preferred configuration, the right hand still being their dominant one (they might sometimes still use the right hand on the lid).^1^ This possibly raises the need for more fine grained measures of hand preference. Gijssels and Casasanto (2020) indeed point out that individual actions vary continuously, rather than categorically, either in the extent of the involvement of each hand (for bimanual actions), or in the extent to which people consistently prefer one hand over the other (for unimanual actions). These kinds of considerations have important implications for experiments on embodied cognition, in that categorical measures might lead to higher rates of type II errors. In the case of the current study, the actions described in the experimental items might have varied continuously both in the strength of the individual preference for a hand configuration, and in the relative workload of the dominant and non-dominant hand. Another explanation for the absence of a mismatch advantage for the left-hand-on-lid group in Experiment 2 lies in the misalignment of motor dimensions in the different movements: one might argue that the lid-turning movement and the container-turning movement do not occur along the same motor dimension, with the lid turning being carried out by means of adduction–abduction movements, and the container-turning via flexion–extension movements; in contrast, turning the experimental knob requires only adduction–abduction movements. The divergence of the container-turning and the knob-turning movements in terms of motor dimensions involved might support the conclusion of a null effect for the left-hand-on-lid participants (as found in Experiment 2, the one with the more robust left-hand-on-lid sample), with the mismatch advantage in Experiment 1 simply reflecting random variation. If the divergence really excludes any kind of overlap between the two movements, they need to be brought under a common denominator at a more abstract level to justify a mismatch advantage: for example, they might have a common directionality marking. Bub and Masson (2010) consider the possibility that the motor resonance in Zwaan and Taylor (2006) reflects the correspondence of only more generic action features, such as direction of motion. There is nonetheless no sufficient evidence available to lean towards the one or the other experiment in terms of credibility of the results for the left-hand-on-lid subgroup, and the issue requires further investigation to reach conclusiveness.

It should be further noted that the results reported in this study are fruit of a rather nonconservative analysis and should, therefore, be interpreted with caution. The factor Frame was dropped from the analyses due to power concerns. We are aware that the embodied account would predict a differential behavior across frames in the first place, and a more conservative analysis including the factor Frame would allow to draw stronger conclusions. At the same time, a test interested in detecting a potentially very small interaction is likely to require a sample size that is simply too large for us to collect. The reported results should, therefore, be seen as providing compatible evidence within the limits of the available resources.

With regard to the issue of the conflicting results of the studies by Claus (2015) and Zwaan and Taylor (2006), the present results do not support the assumption that either the number of characters described nor the different devices are decisive. In fact, they are inconclusive in this respect. However, they suggest that it is necessary to take into account individual differences in action execution. For the study by Claus (2015), the information whether participants have a preference for the right or the left hand on the lid when opening/closing bottles is not available. An account of her mismatch advantage finding in terms of individual differences would presuppose a stable left-hand-on-lid preference for the majority of her participants, which seems unlikely considering our data.

Whereas contrasting versions of the embodied-simulation view of language comprehension differ in their claims, mostly with respect to the strength attributed to the functional role of the simulations for comprehension (Kaup et al., 2015), they all share the underlying assumption that experiential traces are reactivated during language processing. The questions of whether modal experiential traces are the only meaning representations available for language processing and, if not, to what extent they are functionally relevant to comprehension, are still open (Dove, 2009; Zwaan, 2014) and are not tackled in our study. In its most radical interpretation, embodied simulation views assume an equivalence between comprehension and mental simulation, whereby modal traces are the only meaning representations available. One implication of this account is that simulations should be found not only at word-, but also at phrase- and sentence-level. Evidence in this sense remains limited (e.g., Taylor & Zwaan, 2008; Taylor et al., 2008), as it is not straightforwardly possible to disentangle whether the reactivation of sensorimotor traces is driven by experiential higher level mental simulations or mediated by more general linguistic/conceptual associations. In the case of the present study for example, we cannot safely assume the effects to reflect sentence- (or phrase-) level simulations. In fact, although it is unlikely that the effects stem from any single word employed in the sentences, associations might still hold between combinations of words and experiences (such as *open* and *bottle* and the action of rotating the lid counterclockwise) and this could explain our results without the need for modal meaning composition (as discussed in Kaup et al., 2012). On the other hand, studies such as Taylor and Zwaan (2008) and Taylor et al. (2008) point to compatibility effects resulting from modal meaning composition. Taylor and Zwaan (2008) found an action-sentence compatibility effect for action-modifying adverbs (e.g., *On the shelf, he found a closed jar which he opened rapidly*) but not for agent-modifying adverbs (*hungrily* instead of *rapidly*). Taylor et al. (2008) found a compatibility effect for adjectives that allowed for inferences with respect to to the direction of an action that was mentioned earlier in the text (e.g., *He approached the stereo and adjusted the volume. The music was too loud*). At the very least, these results are difficult to explain with simple associations of words and experiences.

As for the assumption of the experiential nature of the traces reactivated during language processing, such an assumption would predict that, if people differ in their experience, accordingly will their simulations (as supported by the findings of Casasanto, 2009; Holt & Beilock, 2006; Lyons et al., 2010). Despite the evidence for the activation of sensorimotor traces during language processing, the majority of the studies do not allow to conclude that these sensorimotor traces are based on the experiences of the individual at hand (Öttl et al., 2017; Casasanto, 2011). Although not providing conclusive evidence, the present findings seem to reflect differences in the reactivation of experiential traces during the simulation process which are brought about by differences in individual experience. The different patterns of results found for the two groups seem to be related to the experiential differences, as self-assessed by the participants. Nevertheless, whereas the interaction effect strongly supports the experiential basis of compatibility effects, it does not guarantee the direct reactivation of motor experiences: the results are additionally compatible with, e.g., the hypothesis that movements might have a directionality marking, as well as with the idea that participants might be simulating the perceptual effects of the movements rather than more straightforwardly reactivating the motor experience itself.

To sum up, the current study addressed the linking of compatibility effects with individual motor experiences. Altogether, the results are suggestive of the potential need to take into account individual experiences in studies on embodied simulation views , which may help truly test the assumptions underlying these views.

## Data Availability

The datasets analysed during the current study are available in the OSF repository, https://osf.io/yr5a3/?view_only=9513f09324ed4e648ab15620d63c1a2a.

## Appendix A: Experimental Items

English translation and German version (in parentheses) of the critical sentences of the experimental items, in clockwise (“closes”) and counterclockwise (“opens”) version.

**Object 1.**

**Clockwise.** Setting: bartender at the end of the shift | She closes an amaretto bottle behind the counter. (Sie schließt eine Amarettoflasche hinter dem Tresen.)

**Counterclockwise.** Setting: bartender at work | He opens an amaretto bottle at the counter. (Er öffnet eine Amarettoflasche an der Theke.)

**Object 2.**

**Clockwise.** Setting: closing a stand at a street festival | He closes a bottle of apple juice at the drinks table. (Er schließt eine Apfelsaftflasche am Getränketisch.)

**Counterclockwise.** Setting: organising a school party | She opens a bottle of apple juice from the fridge. (Sie öffnet eine Apfelsaftflasche aus dem Kühlschrank.)

**Object 3.**

**Clockwise.** Setting: afer feeding a baby | He closes a baby food jar on the park bench. (Er schließt ein Babybreiglas auf der Parkbank.)

**Counterclockwise.** Setting: feeding a baby | She opens a baby food jar on the kitchen counter. (Sie öffnet ein Babybreiglas auf dem Küchentresen.)

**Object 4.**

**Clockwise.** Setting: gas station attendant at the end of the shift | He closes a petrol can at the fuel pump. (Er schließt einen Benzinkanister an der Zapfsäule.)

**Counterclockwise.** Setting: recycling workshop | She opens a petrol can at the workbench. (Sie öffnet einen Bezinkanister an der Werkbank.)

**Object 5.**

**Clockwise.** Setting: cleaning after a party | He closes a bottle of Campari on the living room table. (Er schließt eine Campariflasche auf dem Wohnzimmertisch.)

**Counterclockwise.** Setting: cocktailparty | She opens a bottle of Campari from her house bar. (Sie öffnet eine Campariflasche aus ihrer Hausbar.)

**Object 6.**

**Clockwise.** Setting: after a picnic | She closes a bottle of Coke on her picnic blanket. (Sie schließt eine Colaflasche auf ihrer Picknickdecke.)

**Counterclockwise.** Setting: open-air pool in the summer | He opens a bottle of Coke on the towel. (Er öffnet eine Colaflasche auf dem Badetuch.)

**Object 7.**

**Clockwise.** Setting: car quality control | She closes a gas cap on a truck. (Sie schließt einen Tankdeckel an einem LKW.)

**Counterclockwise.** Setting: oldtimer rallye | He opens a fuel cap on a convertible. (Er öffnet einen Tankdeckel an einem Cabriolet.)

**Object 8.**

**Clockwise.** Setting: making pasta salad | He closes a vinegar bottle over the sink. (Er schließt eine Essigflasche über dem Spülbecken.)

**Counterclockwise.** Setting: pickling beetroots | She opens a vinegar bottle over the preserving pot. (Sie öffnet eine Essigflasche über dem Einmachtopf.)

**Object 9.**

**Clockwise.** Setting: cleaning after childrens’ party | He closes a bottle of Fanta on the dining table. (Er schließt eine Fantaflasche auf dem Esstisch.)

**Counterclockwise.** Setting: barbecue evening | She opens a bottle of Fanta from the cool bag. (Sie öffnet eine Fantaflasche aus der Kühltasche.)

**Object 10.**

**Clockwise.** Setting: after making hot dogs | She closes a cucumber jar on the shelf. (Sie schließt ein Gurkenglas auf der Ablage.)

**Counterclockwise.** Setting: drinking vodka | He opens a cucumber jar from Russia. (Er öffnet ein Gurkenglas aus Russland.)

**Object 11.**

**Clockwise.** Setting: taking care of sick family | He closes a bottle of cough syrup at the medicine cabinet. (Er schließt eine Hustensaftflasche am Arzneischränkchen.)

**Counterclockwise.** Setting: nurse taking care of patients | She opens a bottle of cough syrup from the medicine room. (Sie öffnet eine Hustensaftflasche aus dem Medikamentenraum.)

**Object 12.**

**Clockwise.** Setting: after breakfast | He closes a yogurt jar on the fridge. (Er schließt ein Joghurtglas am Kühlschrank.)

**Counterclockwise.** Setting: making a turkish recipe | She opens a glass of yogurt next to the recipe book. (Sie öffnet ein Joghurtglas neben dem Rezeptbuch.)

**Object 13.**

**Clockwise.** Setting: making salad for the street festival | She closes a mayonnaise tube next to the salad bowl. (Sie schließt eine Mayonnaisetube neben der Salatschüssel.)

**Counterclockwise.** Setting: eating fries | He opens a mayonnaise tube from the sauce stand. (Er öffnet eine Mayonnaisetube aus dem Soßenständer.)

**Object 14.**

**Clockwise.** Setting: feeding newborn cats | She closes a bottle of milk in the shelter kitchen. (Sie schließt ein Milchfläschchen in der Tierheimküche.)

**Counterclockwise.** Setting: working at a newborn unit | He opens a bottle of milk at the sink. (Er öffnet ein Milchfläschchen am Spülbecken.)

**Object 15.**

**Clockwise.** Setting: after baking a fruitcake | She closes a peach glass next to the fridge. (Sie schließt ein Pfirsichglas neben dem Kühlschrank.)

**Counterclockwise.** Setting: making dessert | He opens a peach glass next to the blender. (Er öffnet ein Pfirsichglas neben dem Mixer.)

**Object 16.**

**Clockwise.** Setting: after dinner | He closes a cranberry glass at the dining table. (Er schließt ein Preiselbeerglas am Esstisch.)

**Counterclockwise.** Setting: eating berries | She opens a cranberry glass on the coffee table. (Sie öffnet ein Preiselbeerglas auf dem Couchtisch.)

**Object 17.**

**Clockwise.** Setting: at the bike rental | It closes a tire valve on a tandem. (Sie schließt ein Reifenventil an einem Tandem.)

**Counterclockwise.** Setting: before a bicycle tour | He opens a tire valve on his bike. (Er öffnet ein Reifenventil an seinem Fahrrad.)

**Object 18.**

**Clockwise.** Setting: making sandwiches | He closes a remoulade tube next to the bread box. (Er schließt eine Remouladentube neben dem Brotkasten.)

**Counterclockwise.** Setting: making potato salad | She opens a remoulade tube at the kitchen table. (Sie öffnet eine Remouladentube am Küchentisch.)

**Object 19.**

**Clockwise.** Setting: making grape juice | She closes a bottle of juice on the kitchen table. (Sie schließt eine Saftflasche auf dem Küchentisch.)

**Counterclockwise.** Setting: having a drink after work | He opens a bottle of juice from the bottle shelf. (Er öffnet eine Saftflasche aus dem Flaschenregal.)

**Object 20.**

**Clockwise.** Setting: closing snack stand | She closes a mustard tube next to the cutting board. (Sie schließt eine Senftube neben dem Schneidebrett.)

**Counterclockwise.** Setting: making bavarian white sausages | He opens a mustard tube from his shopping basket. (Er öffnet eine Senftube aus seinem Einkaufskorb.)

**Object 21.**

**Clockwise.** Setting: after a day of renovating a houseboat | He closes a bottle of turpentine on the outer deck. (Er schließt eine Terpentinflasche auf dem Außendeck.)

**Counterclockwise.** Setting: student at the art academy | She opens a bottle of turpentine at the painting table. (Sie öffnet eine Terpentinflasche am Maltisch.)

**Object 22.**

**Clockwise.** Setting: working at the kitchen of a retirement home | She closes a thermos next to the coffee machine. (Sie schließt eine Thermosflasche neben der Kaffeemaschine.)

**Counterclockwise.** Setting: after reaching a mountain summit | He opens a thermos on the wooden bench. (Er öffnet eine Thermosflasche auf der Holzbank.)

**Object 23.**

**Clockwise.** Setting: helping kids brush their teeth | He closes a toothpaste tube over the sink. (Er schließt eine Zahnpastatube über dem Waschbecken.)

**Counterclockwise.** Setting: before bed | She opens a toothpaste tube from the toothbrush glass. (Sie öffnet eine Zahnpastatube aus dem Zahnputzglas.)

**Object 24.**

**Clockwise.** Setting: tidying up the apartment | He closes a cream tube on the edge of the bathtub. (Er schließt eine Cremetube auf dem Badewannenrand.)

**Counterclockwise.** Setting: getting ready in the morning | She opens a cream tube in front of the mirror. (Sie öffnet eine Cremetube vor dem Spiegel.)

## Appendix B: ANOVAs Analysis

The data cleaning for the analysis akin to Claus (2015) was the following: RTs smaller than 100 ms were cut out. Reading times were regressed on the number of characters per frame. A linear regression equation was computed for each participant to predict reading times of the frames of the second sentences of the experimental texts from the number of characters, using the frames of the second sentence of the fillers. All residual reading times smaller than -600 ms were not considered. All remaining residuals deviating more than 2.5 standard deviations from the mean of the corresponding condition (Match x Frame x Subject) were excluded. The results of this analysis are qualitatively equivalent to the results of the main analysis described above (see Tables 1 and 2 for a summary).

**Table 1:** Experiment 1: summary of the analysis akin to Claus (2015)

	By subject	By item
2 (Match) × 3 (Frame)	$N_{1}=79$, $F_{1}(2,156)=0.01$, $p=0.98$, $\eta _{G}^{2}<0.01$	$F_{2}(2,46)=0.005$, $p=0.98$, $\eta _{G}^{2}<0.01$
Frame (overall)	$N_{1}=79$, $F_{1}(2,156)=31.33$, $p<0.001$, $\eta _{G}^{2}=0.18$	$F_{2}(2,46)=61.34$, $p<0.001$, $\eta _{G}^{2}=0.52$
Match (overall)	$N_{1}=79$, $F_{1}(1,78)=0.27$, $p=.61$, $\eta _{G}^{2}<0.01$,	$F_{2}(1,23)=0.27$, $p=0.61$, $\eta _{G}^{2}=<0.01$
Match on Frame 2	$N_{1}=79$, $t_{1}(78)=-0.35$, $p=0.73$	$t_{2}(23)=-0.35$, $p=0.73$
Match on Frame 3	$N_{1}=79$, $t_{1}(78)=-0.25$, $p=0.80$	$t_{2}(23)=-0.22$, $p=0.83$
2 (Match) × 3 (Frame) × 2 (Hand.on.Lid)	$N_{1}=79$, $F_{1}(2,154)=3.25$, $p=0.05$, $\eta _{G}^{2}<0.01$	$F_{2}(2,46)=2.02$, $p=0.16$, $\eta _{G}^{2}=0.01$
2 (Match) × 2 (Hand.on.Lid) on Frame 2	$N_{1}=79$, $F_{1}(1,77)=10.88$, $p<0.01$, $\eta _{G}^{2}=0.02$	$F_{2}(1,23)=6.89$, $p<0.05$, $\eta _{G}^{2}=.06$
2 (Match) × 2 (Hand.on.Lid) on Frame 3	$N_{1}=79$, $F_{1}(1,77)=4.62$, $p<0.05$, $\eta _{G}^{2}<0.01$	$F_{2}(1,23)=3.95$, $p=0.06$, $\eta _{G}^{2}=0.03$
Match on Frame 2 (right-hand-on-lid) (one-tailed)	$N_{1}=56$, $t_{1}(55)=-2.20$, $p<.05$, $d = .29$	$t_{2}(23)=-3.17$, $p<.01$, $d=.65$
Match on Frame 2 (left-hand-on-lid)	$N_{1}=23$, $t_{1}(22)=2.32$, $p<.05$, $d = .48$	$t_{2}(23)=1.79$, $p<.05$, $d=.37$
Match on Frame 3 (right-hand-on-lid)	$N_{1}=56$, $t_{1}(55)=-1.33$, $p=.09$, $d = .18$	$t_{2}(23)=-1.38$, $p=.09$, $d = .28$
Match on Frame 3 (left-hand-on-lid)	$N_{1}=23$, $t_{1}(22)=1.84$, $p<.05$, $d=.38$	$t_{2}(23)=1.45$, $p=.08$, $d=.30$

**Table 2:** Experiment 2: summary of the analysis akin to Claus (2015)

	By subject	By ttem
2 (Match) × 3 (Frame) × 2 (Hand.on.Lid)	$N_{1}=79$, $F_{1}(2, 154)=.30$, $p=.74$, $\eta _{G}^{2}<0.01$	$F_{2}(2, 46)=0.39$, $p=.68$, $\eta _{G}^{2}<0.01$
2 (Match) × 2 (Hand.on.Lid) on Frame 2	$N_{1}=79$, $F_{1}(1,77)=3.52$, $p=0.06$, $\eta _{G}^{2}<0.01$	$F_{2}(1,23)=5.00$, $p<0.05$, $\eta _{G}^{2}=0.03$
2 (Match) × 2 (Hand.on.Lid) on Frame 3	$N_{1}=79$, $F_{1}(1,77)=0.52$, $p=0.47$, $\eta _{G}^{2}<.01$	$F_{2}(1,23)=.67$, $p=0.42$, $\eta _{G}^{2}<.01$
Match on Frame 2 (right-hand-on-lid) (one-tailed)	$N_{1}=43$, $t_{1}(42)=-2.11$, $p<.05$, $d=.32$	$t_{2}(23)=-2.55$, $p<0.01$, $d=0.52$
Match on Frame 2 (left-hand-on-lid)	$N_{1}=36$, $t_{1}(35)=0.33$, $p=0.37$, $d=0.06$	$t_{2}(23)=0.44$, $p=0.33$, $d=0.09$

## Footnotes

1 An informal observation from another of our studies in which we included the Edinburgh Handedness Inventory on a five-point scale (*left hand strongly preferred*, *left hand preferred*, *no preference*, *right hand preferred*, *right hand strongly preferred*) seems to support this claim: for participants classified as right-handers ($N=163$), the ratio of number of answers *right hand strongly preferred*/*right hand preferred* is 6.4 times higher than the ratio *left hand strongly preferred*/*left hand preferred*. This suggests that, when right-handers express a preference for the left hand, this preference is, on average, less strong than when they prefer their dominant hand.
